# Emergence of Shear Bands in Confined Granular Systems: Singularity of the *q*-Statistics

**DOI:** 10.3390/e20110862

**Published:** 2018-11-09

**Authors:** Léo Viallon-Galinier, Gaël Combe, Vincent Richefeu, Allbens Picardi Faria Atman

**Affiliations:** 1 Univ. Grenoble Alpes, CNRS, Grenoble-INP, Institute of Engineering Univ. Grenoble Alpes, 3SR, F-38000 Grenoble, France; 2 Departamento de Física and National Institute of Science and Technology for Complex Systems, CEFET-MG, Belo Horizonte CEP 30510-000, Brazil

**Keywords:** granular materials, displacement fluctuations, *q*-Gaussian, strain localization

## Abstract

The statistics of grain displacements probability distribution function (*pdf*) during the shear of a granular medium displays an unusual dependence with the shear increment upscaling as recently evinced (see “experimental validation of a nonextensive scaling law in confined granular media”). Basically, the *pdf* of grain displacements has clear nonextensive (*q*-Gaussian) features at small scales, but approaches to Gaussian characteristics at large shear window scales—the *g*ranulence effect. Here, we extend this analysis studying a larger system (more grains considered in the experimental setup), which exhibits a severe shear band fault during the macroscopic straining. We calculate the *pdf* of grain displacements and the dependency of the *q*-statistics with the shear increment. This analysis has shown a singular behavior of *q* at large scales, displaying a non-monotonic dependence with the shear increment. By means of an independent image analysis, we demonstrate that this singular non-monotonicity could be associated with the emergence of a shear band within the confined system. We show that the exact point where the *q*-value inverts its tendency coincides with the emergence of a giant percolation cluster along the system, caused by the shear band. We believe that this original approach using Statistical Mechanics tools to identify shear bands can be a very useful piece to solve the complex puzzle of the rheology of dense granular systems.

## 1. Introduction

The knowledge of the probability distribution function (*pdf*) for the velocities of systems composed of particles is an essential information to assess the nature of microscopic interactions governing such systems. A fundamental result of Statistical Mechanics concerns exactly the velocities *pdf* for a collection of particles confined into a box, under the molecular chaos hypothesis, the celebrated Maxwell–Boltzmann distribution [1]. In addition, under this assumption, collisions between particles occur randomly, and it is thus possible to anticipate that the fluctuations in the particle velocities will display a *pdf* with Gaussian features. This is a consequence of the central limit theorem (*clt*), once the particle velocity at a given time *t* is the result of a sequence of random events (collisions), leading to a Gaussian distribution no matter how the events are distributed themselves.

These results are very robust and most of the researchers take them as a priori for data analysis. However, in nature, several systems exhibit long range inter-particle interactions, a feature which breaks one of the postulates of the molecular chaos hypothesis. In those systems, it is common to observe important deviations from Gaussian *pdf* in the particle-fluctuation statistics. Tsallis and collaborators [2] have shown that it is possible to extend the *clt* to consider systems which exhibit long range or dissipative interactions by introducing the *q*-Gaussian distribution [3,4], where the *q* is an additional parameter that allows for controlling the weight of the *pdf* tail. In the limit q=1, the Gaussian distribution is recovered, and, for q≠1 heavy or light tails—platycurtic or leptokurtic distributions, respectively—are obtained. Over the last few years, Tsallis statistics were proven to successfully describe several systems in high energy literature [5,6], magnetic systems [7,8], dissipative systems [9] and, remarkably, several deviations from the Gaussian-like behavior were also reported in confined granular systems [10,11,12].

Recently, studies with confined granular systems under shear introduced an additional problem assessing the displacement fluctuations *pdf* of a given system (the displacement fluctuations correspond to the displacement deviations from the mean field). The authors have demonstrated that the *pdfs* of particle fluctuations are strongly dependent on the strain increment (akin to time scale) considered in between the events [13,14]. The *pdf* of fluctuations evolves from Gaussian-like, when large scales were considered, to heavy tail distribution for small scales, which are well described by *q*-Gaussian up to five decades for the magnitude of the fluctuations of displacements [15]. This unexpected result is indeed quite robust as it was used to verify the Tsallis–Bukman scaling law [14,16] which relates the *q*-value obtained from fluctuation *pdf* with the diffusion exponent α, related to the anomalous diffusion of the grains [17]. Tsallis and Bukman have demonstrated that when long-range correlations are present in the system, leading to an anomalous behavior in the path drift of particles, the corresponding fluctuation *pdf* exhibits heavy tails, diverging from the Gaussian-like shape. The predicted relation between these exponents, the Tsallis-Bukman scaling law α=2/(3−q), was verified experimentally by some works [11] but only at specific points. The first experimental verification of this law for a wide range of points was made by Combe and collaborators for a confined granular system under shearing [14]. It is noteworthy that the measures of *q* and α were completely independent, contributing to the strength of this result.

Indeed, the shear experiment with heterogeneous granular media is an ideal system to study the particle displacement fluctuations. It imposes a homogeneous deformation field over the system, but, due to the heterogeneous nature of the grains, the steric exclusion imposed by the rigidity of particles implies a wide range of magnitude to the grain fluctuations along the experiment (see Supplementary Material in [14]). Thus, a detailed evaluation of the *pdf* of grain fluctuations is essential to a proper analysis of the microscopic interactions going on in the system. In this work, we made a step further in this direction by considering a new granular system with a different size grading but applying the same methodology as in [14]. The different grading curve employed in the present study resulted in a significant increase of the packing fraction of the system. Not surprisingly, we observed an earlier development of *shear bands* during the experiments; they are zones of concentration of shear strain which are stable and can span the whole system. After a careful image analysis, we have concluded that it is possible to associate the emergence of a shear band with a sudden change in the value of the *q* parameter measured as a function of the time scale. This singular behavior of *q* associated with a localization event in a confined granular system, never reported before, opens a new perspective to use Statistical Mechanics tools as estimators for the emergence of shear bands or even other analogous phenomena in granular systems and heterogeneous media in general.

The paper is structured as follows: after this brief introduction, we present the experimental methodology and the details of the image analysis procedures. Next, we discuss the shear band mechanics and our results under the *q*-statistic viewpoint. An image analysis of the particle fluctuations considering percolation concepts is then discussed and compared with the preceding analysis. We close the paper presenting our conclusions and some perspectives for future works.

## 2. Materials and Methods

### 2.1. Experimental Setup

**Figure 1:**
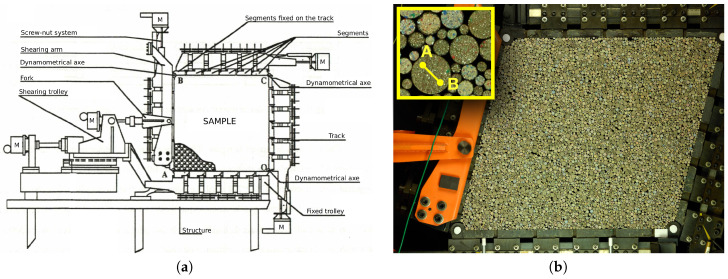
(**a**) sketch of 1γ2ε apparatus; (**b**) granular sample of 11,975 *Schneebeli* rods sheared in 1γ2ε. The inset is a zoom on a few rods of five different diameters from 3.09 mm to 12.14 mm. The example of a segment [AB] used to proceed the *DIC* accuracy.

In this study, a 2D analogous granular material has been sheared with the 1γ2ε shear apparatus [18,19] at Laboratoire 3SR in Grenoble, France, Figure 1. The 1γ2ε device is mainly a deformable parallelogram, which deformation is controlled by five electric motors (two synchronized for height, two for width and a last one for shear). Forces sensors installed at the corners of the parallelogram are used to compute the stress applied to the granular material. Thanks to a retro-action loop, the size of the parallelogram can be modified to impose a stress. All possible 2D strain and/or stress path can be imposed to the granular media: uniaxial compression, isotopic compression, shear, etc. This apparatus dedicated to granular materials have been used for research on various topics, such as the sensitivity of the mechanical behavior to the stress loading path [19]; the influence of grain shape [20] with the use of polygonal grains, mixed or not with round grains; the micro-mechanical investigations of the effect of a highly deformable intruder in a confined granular media [21]; and the grain fluctuations in a sheared granular assembly [14,22].

The granular matter used in 1γ2ε is a 2D analogous material called Schneebeli rods [23]. These rollers of 6 cm long, named grains hereafter, are painted on their visible face with a speckle to be followed during the test (see inset of the Figure 1b). For all experiments presented in this paper, the granular samples were made of 11,975 grains with five different diameter sizes: 6656 grains of 3.09 mm, 2810 grains of 4.95 mm, 1459 grains of 7.09 mm, 833 grains of 9.06 mm and 217 grains of 12.14 mm. Two experimental tests were performed:

- vertical compression with an imposed vertical velocity $v_{0}$ (constant imposed vertical strain rate $\dot{\varepsilon_{y}}$) with the lateral stress kept constant ($\sigma_{0}=50$ kPa)—also called *biaxial vertical compression* or *biaxial test*;
- and shear test, for which 1γ2ε parallelogram is tilted with an imposed shear rate $\dot{\gamma }$. During the shear, the horizontal size of the sample is constant and a constant normal stress $\sigma_{0}$ in imposed on the top of the sample. This test is also referred to as *simple shear test*.

**Figure 2:**
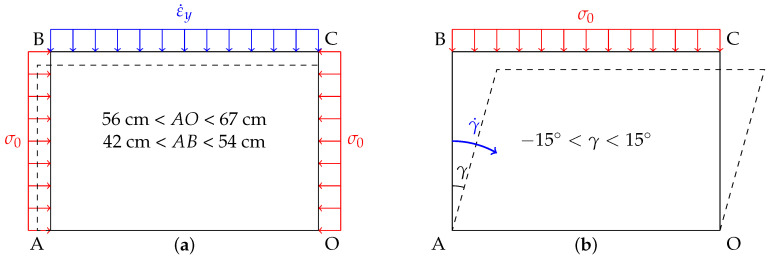
Sketches of the two classical 2D tests realized: (**a**) a biaxial test and (**b**) a simple shear test. The imposed strain rates (${\dot{\varepsilon }}_{y}$ or $\dot{\gamma }$) are displayed in blue, and the imposed stresses ($\sigma_{0}=50$ kPa) are displayed in red. The straining ranges (extension lengths and tilting angles) permitted by the device 1γ2ε are also provided.

The sketches of the principle of these tests are presented in Figure 2. The imposed wall-velocity was always sufficiently low to consider that the evolution of the granular assemblies is quasi-static, which practically means that the overall mechanical response is not affected by possible adventitious “jumps” of some particles that would move dynamically; such dynamic effect can be neglected. This could be ensured considering the dimensionless *inertial number* [24,25] $I=\dot{\gamma }d/\sqrt{\sigma_{0}\rho }$ where $\sigma_{0}$ is the confinement pressure, *d* is the mean grain diameter and $\dot{\gamma }$ is the imposed shear rate. This inertial number measures the ratio of inertial forces of grains to imposed forces. It can also be interpreted as the time scale for grains to rearrange due to the confining stress, to the time scale for deformation by the flow. A third interpretation is the ratio of collisional stress to total stress. In terms of rheology, the so called μ(I) model discussed in [25] allows for a clear partition of the flowing regimes as a function of *I*; and it is observed and well established that $I<10^{-3}$ always satisfies the fact that, in a steady flow, both the internal friction μ and the volume change (solid fraction) are not dependent of *I*. The largest inertial number used in this paper is about $10^{-6}$, which is well below $10^{-3}$ and allows us to safely make the assumption of quasistatic flow.

### 2.2. Assessment of the Full Grain-Kinematics

Digital Image Correlation (*DIC*) is used to track each grain along all the photographs. This technique was firstly suggested in [26] and largely developed since the beginning of the century to provide kinematics fields from images (displacement, strain, accelerations, etc.) in continuous materials (metals, polymers, concrete, etc.). In our case, the goal is not to determine strain fields from *local mean* displacements of an amount of grains but to follow the motion of each individual grain (supposed to be rigid). The quantities to be determined are the displacement (components Δx and Δy) and the rotation (Δθ) relative to the first image. This is very different from classical applications of *DIC* as grains could move erratically and significantly between two successive images (especially in case of grain rearrangements), so the variations Δx, Δy and Δθ are not necessarily small or continuous in space. Specific tools are thus needed to follow individual grains—without losing any of them and with high precision of the tracked motions—in granular materials. The tool Tracker [27] has been developed exactly for this purpose.

The basis of the *DIC* is to find the displacement of a pattern (subset of the reference image) on all images. The best pixel position is chosen with the best correlation coefficient φ, which is the *zero-mean normalized cross-correlation* (ZNCC) coefficient. First, all positions and rotations are tested at pixels scale in a ranged neighborhood of last known position of the grain by assuming that grains do not move a lot during the 5 s between successive photographs, typically around 1 pixel. The recognition is supposed to be correct if φ is not under a given threshold (φ>0.8); otherwise, the neighborhood ranges are enlarged. At this point, the pixel position is given by integers and the rotation is *quantized* due to the discrete nature of the signal. To refine the positions and rotations at a sub-pixel resolution, the signal from second image is made continuous thanks to a bi-cubic interpolation of the gray levels, and the best values of (Δx, Δy, Δθ) are found with a sub-pixel precision by minimizing (1−φ); Powell’s conjugate direction method [28] is used to this end.

For Schneebeli rods, circular patterns centered on grains are used; their diameters are 80% of the tracked grain diameters to avoid local deformations due to contact to be in the correlation area. Localization of already identified grains are also used to reduce the search area and make the first stage of the correlation faster. After the grain tracking, the positions are corrected to account for image distortion with the Brown–Conrady model [29]. The scaling of images is done on the first images with two points whose distance is known at the beginning of the test. The movement of the camera is corrected with the help of a set of points which are not supposed to move on the frame of the 1γ2ε apparatus and covered by a speckle. Grains with an insufficient correlation coefficient (under 0.7) are removed from the data by checking that they remain very few.

In all photographs, it is also possible to measure the mean stream, following the four corners of the 1γ2ε, which were also painted with a speckle. Finally, each grain comes with an actual motion and the affine motion it would have if it obeys a homogeneous deformation gradient.

### 2.3. Accuracy of Tracked Kinematics

**Figure 3:**
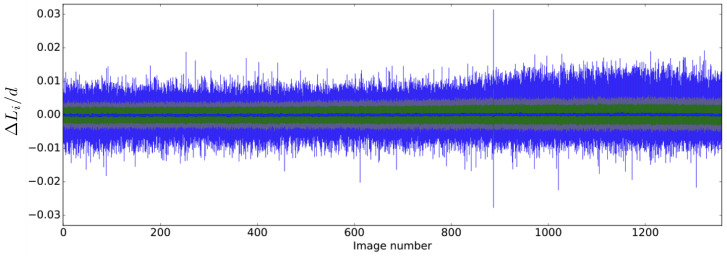
Segment length errors (relative to the mean diameter) over the photograph shots. Mean error $\langle \Delta L_{i}\rangle /d$ (blue points close to zero), standard deviation (green area), 90th percentile (gray area) and total range of errors (blue area) for all segments of each image since the first one (taken as reference). The set contained 434 segments.

Accuracies of measurements of grain kinematics must take into account the full measurement chain, from the camera lens to the *DIC* software. In particular, but not exclusively, inaccuracies come from the lens distortion, the Charge-Coupled Device (CCD) sensor of the camera (that blur the image to avoid Moiré effects), the algorithm used for demosaicing the image (Bayer matrix), the quality of the speckle painted on each grain and the degree of complexity of the function of interpolation of the image (to obtain sub-pixel measurements). A way to assess a reliable measurement of the error on the kinematics is to grab every source of error together. Considering that the grains are perfectly rigid, any internal distance should remain constant whatever the strain/stress path imposed to the granular sample, i.e., whatever the displacements and the rotations of the grains. To test this, the two extremities *A* and *B* of several segments are tracked together with the translation and rotation of all grains in the sample; see inset in Figure 1. Considering the first digital image as a reference, the initial lengths $L_{i}^{0}$ of the segments *i* should not change for the other images. Therefore, the variations of length $\Delta L_{i}\left(N\right)=L_{i}^{N}-L_{i}^{0}$ as a function of the *N*th image provides an estimation of the kinematics error. The average errors $\langle \Delta L_{i}\rangle \left(N\right)$ for each photograph were computed from 434 segments over more than 1300 photographs that have been shot during a shear test (Figure 3) for which the maximum grain displacement was about 10 cm.

In this example, it is possible to observe a standard deviation of $\Delta L_{i}$ in the range ±0.2% of the mean-diameter *d* with a nearly null average error. The distribution of errors $\Delta L_{i}$ follows a Gaussian function that remains nearly the same for all photographs. The maximum error is on the order of ∼1% of the mean diameter, and it goes exceptionally to $\Delta L_{i}/d=3\%$ (this appended actually only one time because of an unintentional kick in the camera). In view of the number of grains in each photograph, this high level of accuracy was made possible thanks to a 80 MPixels digital Camera and a particular care to circumvent the possible sources of inaccuracy (correction of lens distortion and camera parallax, check of pattern quality, choice of the debayering algorithm, careful choice of the interpolation function for sub-pixel gauging, uniform lighting, etc).

### 2.4. Fluctuation of the Displacements

In a granular packing, a grain trajectory does not necessarily follow the homogeneous displacement field imposed at the macroscopic level (sample scale). This is due to geometrical constraints at the grain scale (steric exclusion)—grains are not able to displace as continuum mechanics dictates that they should. The fluctuation of displacement can be defined as the difference between the actual displacement δr—measured with *DIC*—and the displacement that the same point would have if it had obeyed a homogeneous strain (equal to the imposed macroscopic one) $\delta r^{⋆}$.

It is useful to introduce a dimensionless fluctuation *v* as a ratio between the physical displacement fluctuation and the typical length Δεd where Δε is a homogeneous macroscopic strain and *d* is the mean diameter of the grains:

(1) $$v=\frac{\delta r-\delta r^{⋆}}{\Delta \varepsilon \;d}.$$

Depending on the test, Δε can be either an increment of shear strain (variation of the tilt angle) Δγ or the vertical strain $\Delta \varepsilon_{y}$. The 1γ2ε apparatus was designed so that the sample is disposed vertically; that is to say, the gravity acts on the *y* component of our images which could introduce a bias in the fluctuations. To avoid this effect as much as possible, it has been chosen to study mainly the *x* coordinate of the fluctuations, $v_{x}$.

### 2.5. Local Strains

**Figure 4:**
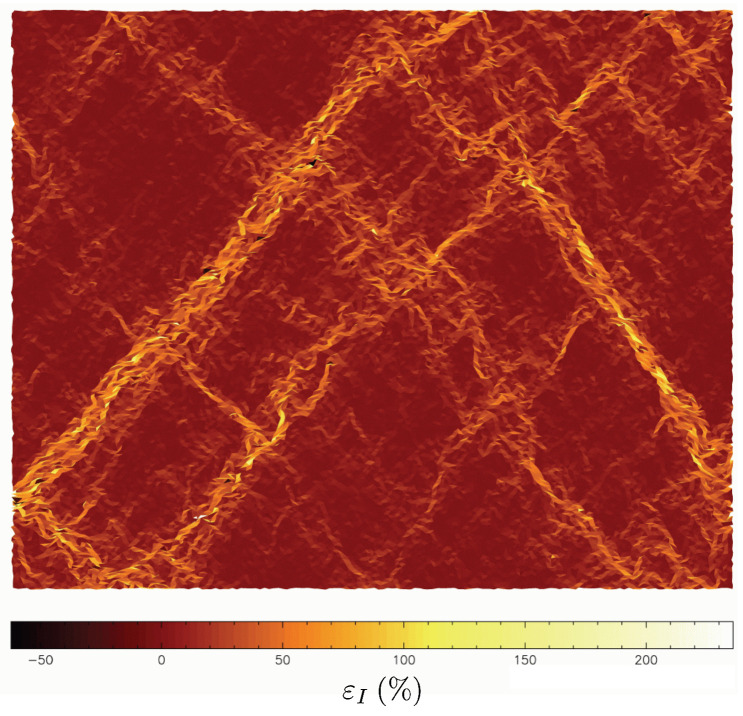
An example of strain localization map ($\varepsilon_{I}$) computed from the definition given in Section 2.5, for a *DEM* assembly of 22,500 particles undergoing a vertical compression (imposed vertical strain rate while an horizontal stress is kept constant).

A local two-dimensional strain tensor can be defined using the Delaunay triangulation of grain centers, where displacements are known. A mean strain tensor for each triangle is given thanks to the Stokes theorem as the contour integral:

(2) $$\underline{\underline{\varepsilon }}=\frac{1}{S}\int_{S}\nabla \underline{u}\;dS=\frac{1}{S}\oint_{\ell }\underline{u}⊗\underline{n}\;d\ell ,$$

where $\underline{u}$ is the displacement vector at the boundary of the triangle, $\underline{n}$ the outgoing normal vector, *S* the triangle surface and *ℓ* the contour length [19]. From the tensor $\underline{\underline{\varepsilon }}$, it is possible to use all the components to highlight local/microscopic strains. However, a convenient way to study the strain localization in granular material is basically to focus on two invariants of the strain: the volumetric strain $\varepsilon_{\mathrm{vol}}=\mathrm{trace}\;\left(\underline{\underline{\varepsilon }}\right)=\varepsilon_{I}+\varepsilon_{II}$ or the shear intensity $\varepsilon_{D}=\varepsilon_{I}-\varepsilon_{II}$, where $\varepsilon_{I}\geq \varepsilon_{II}$ are the two eigenvalues of $\underline{\underline{\varepsilon }}$. It should be noticed that the chosen sign convention is such that a strain component is positive when lengths reduce. Figure 4 gives an example of a shear band network in a granular material subjected to a biaxial compression.

Hereafter, for a sake of simplicity, strain localization will generally be illustrated by means of $\varepsilon_{D}$ which has the advantage of being always positive or equal to zero.

## 3. Shear Band Mechanism

**Figure 5:**
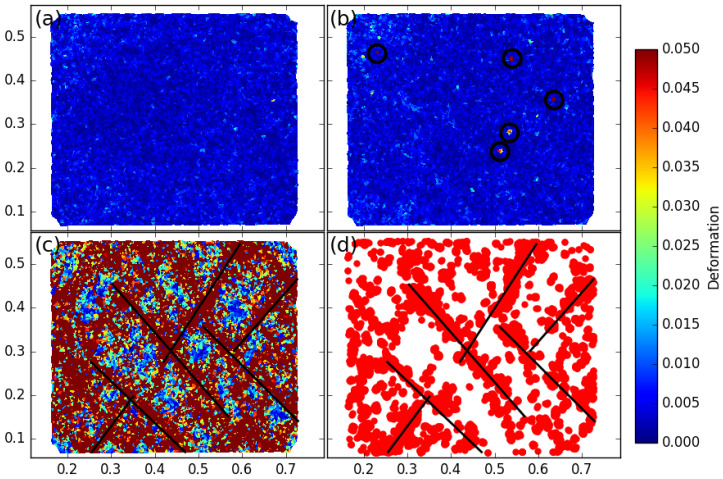
Vertical biaxial compression: field of local shear intensities $\Delta \varepsilon_{D}$ for different macroscopic strain increments $\Delta \varepsilon_{y}$ starting from a state where shear bands are already established in the sample. (**a**) field of local $\Delta \varepsilon_{D}$ for $\Delta \varepsilon_{y}=0.015\%$; (**b**) another case with $\Delta \varepsilon_{y}=0.015\%$ for which some Delaunay triangles present local peaks of $\Delta \varepsilon_{D}$ (highlighted with circles); (**c**) shear intensity map for $\Delta \varepsilon_{y}=2\%$ showing shear bands in the material (some of them are highlighted by lines); (**d**) the localization of local strain peaks observed for increments $\Delta \varepsilon_{y}=0.015\%$, aggregated over 2% of macroscopic strain; the lines are reported from the shear bands of (**c**).

When plasticity occurs in a heterogeneous material submitted to a deviatoric loading, strains can concentrate in specific zones, and their locations are generally impossible to predict. These specific zones are zones where the imposed strains on the sample (macroscopic scale) concentrate locally by making patterns in the form of bands (see Figure 4). These bands are termed *shear bands* since they are the places where the microscopic shear strain over-expresses, the rest of the volume being weakly deformed compared with the mean strain. For dense granular material, it has been shown that strain localization is a key mechanism of plasticity evolution. These shear bands are experimentally observed either for sand [30], for analogous granular material like *Schneebeli* materials [19,31] and also for virtual granular models like those used in DEM (Discrete Element Model) [21], or double scale FEM × DEM (Finite Element Model at macroscopic scale, and Discrete Element Model at the microscopic scale) [32]. It might be important to stress that shear bands are steady with fixed positions and orientation, but they can appear for different shear strain γ during the loading; they, however, never decline for monotonic loading. The emergence of shear bands can be pictured by keeping in mind that the zones of concentrated shear-strain form straight bands (typically 10 to 20 particle diameters in width), they can be multiple and they can be reflected at the sample boundaries (like in the Figure 4 where shearbands reflect on the rigid boundaries). For high shear strain levels, the map of strain anisotropy, as observable in Figure 4 and Figure 5 gives the false impression of locally varying inhomogeneities, but they are actually several straight shear bands having the same inclination (or reflected inclination) that intersect one another. When a granular material is submitted to an external loading path, shear bands do not appear instantaneously. Whereas the orientation of shearband can be more or less predicted by the Rankine’s earth-pressure theory, during the past 50 years, many research works tried to explore and to understand how shear bands appear and develop. Very recently, new progress in the *DIC* technique and with the improvement of very high resolution of digital cameras helps to observe that shear banding can occurs well before reaching the maximum strength of the granular assembly [33]. However, important questions remain: why and how do the shear bands appear?

We focus here on the so-called vertical biaxial compression test. An imposed vertical strain increases with a constant rate all along the compression. This strain rate is such that the sample deforms in a quasi-static regime. At the end of the test, the macroscopic vertical strain can reach $\varepsilon_{y}=10\;\%$ or more. After a given macroscopic vertical strain $\varepsilon_{y}$ (typically 2%), a network of oblique shear bands can already be observed. Strain localized zones can be plotted and identified for a given imposed macroscopic strain increment $\Delta \varepsilon_{y}$ using various local indicators like the increments of the second invariant $\Delta \varepsilon_{D}$ (shear intensity). Strain field of $\Delta \varepsilon_{D}$ have been computed for different macroscopic strain increments $\Delta \varepsilon_{y}$ from a macroscopic strain-level for which the shear bands have already started to develop, $\varepsilon_{y}=0.02$. The first striking point is that, when plotting the map of $\Delta \varepsilon_{D}$ for a very small increment $\Delta \varepsilon_{y}=0.01\%$, the shear bands are not apparent, as shown in Figure 5a. However, sometimes, for such very low strain increments, some concentrated local strain peaks occur (Figure 5b). It is possible to observe fully developed shear bands only for increments of strain greater or equal to 2%, as shown in Figure 5c.

It is observed in Figure 5c that the rare local peaks of $\Delta \varepsilon_{D}$ (computed for the low strain increment, Figure 5d) aggregated in the course of time reconstruct the picture of shear bands. Thus, the concept of shear bands seem to be relevant only when a sufficient macroscopic increment of strain is reached. This separates two regimes in terms of $\Delta \varepsilon_{D}$: when the macroscopic increment of strain is small, no extended localization zone appears except some very local microscopic events with no manifest correlation in space; when the macroscopic strain reaches a sufficiently large value, the imposed macroscopic strain localizes into shear bands that percolate throughout the granular sample.

## 4. Shear Bands: Viewpoint of q-Statistics

### 4.1. q-Gaussian Statistics of Displacement Fluctuations

**Figure 6:**
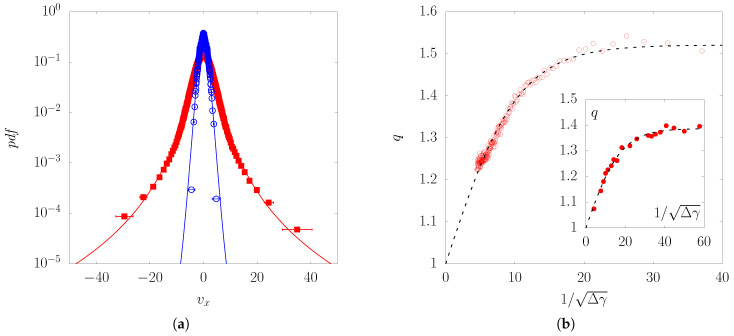
(**a**) probability density function of the horizontal component of the displacement fluctuation $v_{x}$, Equation (1), for two values of strain increment Δγ in case of a simple shear test on a sample of around 2000 rods in the device 1γ2ε. Symbols are experimental inputs (red squares: $\Delta \gamma =2\times 10^{-3}$, blue light circles: $\Delta \varepsilon =10^{-1}$). The curves are *q*-Gaussian fits (red: q=1.51, blue: q=1.16); (**b**) evolution of the parameter *q* with respect to the strain increment Δγ. Inset: same analysis from data obtained by means of DEM on an assembly of 22,500 grains.

When the strain is quite homogeneous throughout a deformed sample, the *pdf* of the normalized displacement fluctuations follows a *q*-Gaussian. This was shown in [14] with a massive amount of experimental data, but with systems with much less particles. A very clear broadening of the fluctuation *pdfs* was observed as the strain increment Δε was increased, [15]. Figure 6a provides an illustration of such broadening.

The dependence of the *q*-exponent with the strain increment Δε used to calculate the fluctuations is shown in Figure 6b, for experimental and numerical simulation data (the inset of the figure shows the data issued from *DEM* simulations). Two remarkable features can be noticed with this plot:

- In the limit of large strain increments (i.e., when the abscissa goes to zero), *q* tends to 1. In other words, the distribution tends to become a Gaussian when Δε→∞. Some DEM simulations made it possible to test larger values of Δε, which confirm the limit q→1 (this data is shown in the inset of Figure 6b). This is exactly what is expected for this limit, once the particles typically experience several contact losses/gains and rearrangements, approaching the molecular chaos hypothesis.
- At the other limit, for vanishing strain increment Δε, the *q*-value attains a plateau that can be interpreted as resulting from long range correlations imposed by the force chains at this short displacement (or time) scale.

These features were observed both in experiments and also in a large number of *DEM* simulations [14], which make the observations very robust provided that the assumption of strain homogeneity is not jeopardized too much.

### 4.2. Evolution of q with an Emergence of Shear Bands

The fluctuations have been defined by assuming an affine field of displacements (or equivalently a homogeneous field of strain) as expressed in Equation (1). However, as soon as strain localization has a well-defined onset anywhere in the sample, the mean field of strains is not a representative quantity anymore that reflects what actually happen. In spite of strain localization, the computation of displacement fluctuations using Equation (1) remains possible, but its physical interpretation is made more complex. Still, this measure is expected to continue to be of interest, as is the relying *q*-statistical description.

**Figure 7:**
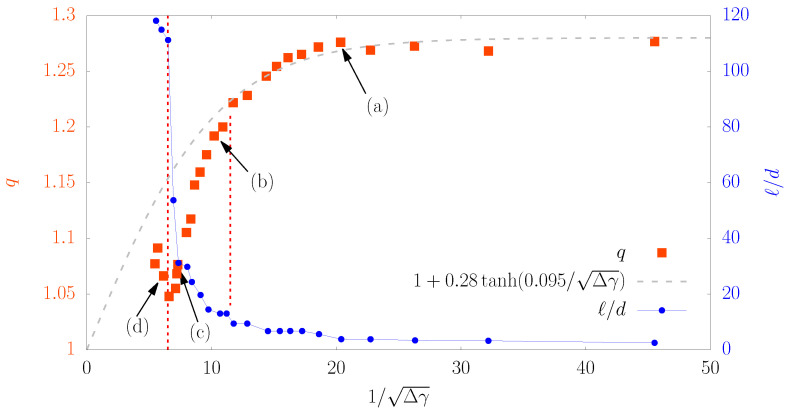
Evolution of the *q*-exponent of the *pdfs* of displacement fluctuations, and of the maximal length of shear bands ℓ/d (number of mean diameters), when the shear strain increment Δγ is increased (from right to left). The gray dashed line serves as guide to the eyes; the “tanh” function has no particular meaning excepted that it tends to 1 when Δγ→∞ and it saturates at a plateau-value when Δγ→0. The vertical dashed lines indicate two transitions of both curves (*q* and ℓ/d): from right to left, the first transition corresponds to the point where *q* starts to derive from the route to 1 (“tanh” function) and ℓ/d starts to take off; the second transition corresponds to the surge of both *q* correlated with the percolation of the shear band throughout the sample.

The idea here is to study the evolution of the *q*-exponent when the strain increment is increased, in the case of a clear localization of the strains through shear bands within the granular material. For this purpose, a shear test was carried out (see the sketch of such loading in Figure 2b). This loading path was preferred because shear bands appear more progressively than with a biaxial compression, so their studies are made easier. The evolution of the *q*-parameter regarding the (inversed squared) increment of deformation is presented in Figure 7.

When the macroscopic strain increment (here a shear strain increment Δγ) is sufficiently small, the localization is not perceptible and similar observations than those of Section 1 can be drawn: a transition from an invariant value of *q* to a regime where *q* starts to go to 1 can be inferred for $1/\sqrt{\Delta \gamma }>12$ (which is Δγ<0.7%). However, when localization initiates (right vertical dashed line in Figure 7), an inflexion of the evolution of *q* drifts from the route towards 1. A third phase can be noticed when Δγ is even more increased for $1/\sqrt{\Delta \gamma }<7$ (which is Δγ > 2%): *q* increases suddenly, which highlights a new type of interaction in the grain displacements.

### 4.3. Link with the Shear Bands

**Figure 8:**
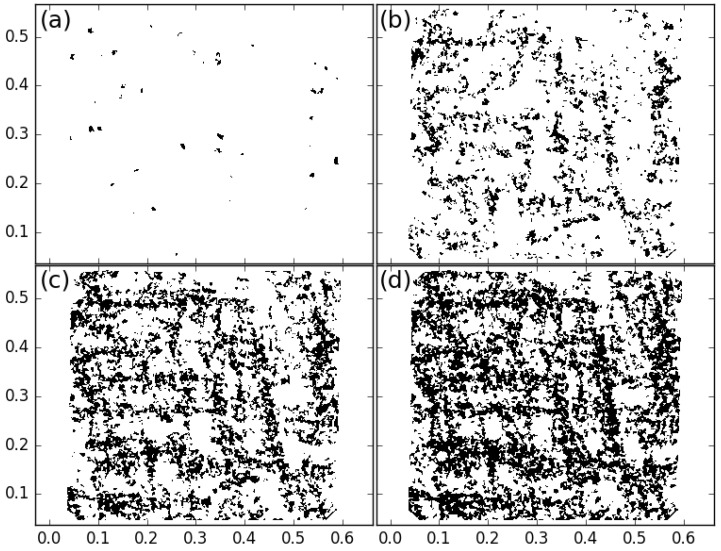
Shear intensity map segmented with a threshold $\Delta \varepsilon_{D}^{0}=3.6\%$. The 100th image of the test is used as a reference to compute strain tensors; the strain increments are (**a**) Δγ=0.23%; (**b**) Δγ=0.94%; (**c**) Δγ=1.67% and (**d**) Δγ=2.4%.

The shear bands can be visualized for different shear increments Δγ in Figure 8, where the shear intensities $\Delta \varepsilon_{D}$ have been segmented thanks to a chosen threshold value $\Delta \varepsilon_{D}^{0}$: black zones correspond to $\Delta \varepsilon_{D}\geq \Delta \varepsilon_{D}^{0}$ and white zones correspond to $\Delta \varepsilon_{D}<\Delta \varepsilon_{D}^{0}$. The threshold value $\Delta \varepsilon_{D}^{0}$ have been chosen as the total macroscopic shear strain corresponding to the maximum achieved tilt, which is $\Delta \varepsilon_{D}^{0}=3.6\%$, and we checked that changing this value in the range 2.4% to 4.1% had no significant influence on the observations presented below.

It can be noticed in these maps that, for lower strain increments (Figure 8a), the bands are not continuous at all, the highly sheared areas are very fragmented. For larger strain increments (Figure 8b,c), these areas tend to aggregate in the form of bands, and, in Figure 8d, the bands seem to be continuous from one border of the sample to an other. These four maps are spotted in Figure 7 so that the relationship with the *q*-values can be done.

To better relate the growth of shear bands with the *q*-parameters, a new indicator is introduced: the maximal length *ℓ* of the shear bands. For each contiguous area of high shear strain (in black), the length *ℓ* is defined as the longest distance joining two grain centers belonging to the same area, using the infinite norm (that is the maximal amplitude over *x* or *y*). The indicator is then the largest length for all contiguous area of high shear strain on the map. The evolution of this maximal length, expressed in number of mean diameters ℓ/d, is plotted as a function of $1/\sqrt{\Delta \gamma }$ in Figure 7 in order to draw a parallel with the evolution of *q*. For smallest strain increments (right of abscissa), *ℓ* is of the order of a few diameters, which indicates that a few grains are involved and thus these “shear bands” are actually local rearrangements. A faster evolution of *ℓ* can then be noticed with an inflexion point corresponding nearly at the start of *q* drift. Finally, unlike what the maps in Figure 8 may suggest, the evolution of the length of the shear bands is not steady. A clear transition arises with length of shear bands reaching suddenly the sample size. This transition occurs precisely for the same strain increment that the transition observed with the *q*-parameter in Figure 7. This latter observation is not sensitive to threshold value employed in the analysis, which makes it very robust.

Thus, the raise of the *q*-parameter for largest increments of deformation could be assigned to the percolation of shear bands, as it occurs exactly when the shear band extension reaches the sample size. In addition, we found a linear dependence between *q* and *ℓ* as the shear band length grows (data not shown), with a negative linear coefficient up to the percolation transition. This is a strong evidence that these two parameters are (anti-)correlated and, thus, both can be used as order parameter for the transition. The possibility to anticipate the shear band formation by means of a direct measure over the system is a very sound tool, and here we report very promising results in this direction.

## 5. Conclusions

The nature of grain displacement fluctuations in confined granular systems under shear was investigated by means of statistical mechanics tools. We extend the previous work that demonstrated an unusual dependence of the nonextensive parameter *q* with the shear strain window scale by characterizing the emergence of a shear band along the system. Our results have shown that the *q*-parameter displays a singular non-monotonic behavior at large scales. Performing an independent image analysis, we identify the emergence of a giant percolation cluster in the system associated with the shear band localization. By comparing the behavior of the percolation order parameter and the *q*-parameter dependence with the shear strain window scales has evinced a remarkable correlation between the emergence of the shear band along the system and the sudden change in the *q*-value. This remarkable observation obtained by an original approach with non-extensive statistical mechanics tool has great potential to impact the research field and also to other areas that deal with similar systems.

We expect to perform more analysis concerning the character of the displacement fluctuations inside the shear bands themselves (like in [34]) by defining the displacement fluctuations relative to the local mean displacements instead of the global one. It is also planned to explore larger systems (with more grains) and slower loadings (lower inertial numbers) in future investigations. Since non-monotonic loadings involve complex transitions [35,36], the features of displacement fluctuations will also be investigated by considering loading cycles around an undeformed state with increasing amplitude. We believe that the approach introduced here has the potential to reveal interesting findings and new perspectives regarding the very special character of grain kinematics in confined granular systems.

## Abbreviations

The following abbreviations are used in this manuscript:

*pdf*	Probability Density Function
*clt*	Central Limit Theorem
*DIC*	Digital Image Correlation
*DEM*	Discrete Element Model
*FEM*	Finite Element Model
